# State-Based and Demographic Variation in Parent-Reported Medication Rates for Attention-Deficit/Hyperactivity Disorder, 2007–2008

**DOI:** 10.5888/pcd9.120073

**Published:** 2013-01-24

**Authors:** Susanna N. Visser, Stephen J. Blumberg, Melissa L. Danielson, Rebecca H. Bitsko, Michael D. Kogan

**Affiliations:** Author Affiliations: Stephen J. Blumberg, Melissa L. Danielson, Rebecca H. Bitsko, Centers for Disease Control and Prevention, Atlanta, Georgia; Michael D. Kogan, Health Resources and Services Administration, Rockville, Maryland.

## Abstract

Medication is the most effective treatment of attention-deficit/hyperactivity disorder (ADHD), a common neurobehavioral disorder of childhood. We used data from the 2007-2008 National Survey of Children’s Health to calculate weighted estimates of parent-reported ADHD and medication treatment among US children aged 4 to 17 years, by state and sex-stratified age. State-based rates of ADHD medication treatment ranged from 33% in Nevada to 79% in Mississippi; rates of medicated ADHD were higher among boys than girls at every age. State-based investigations of ADHD medication treatment factors are needed, and our findings may inform these public health efforts.

## Objective

Attention-deficit/hyperactivity disorder (ADHD) is a common neurobehavioral disorder of childhood (1); 4.1 million (7.2%) US school-aged children have a current diagnosis (2). Medication is the single most effective treatment (3,4), and monitoring ADHD treatment patterns is important for improving treatment in this population. We recently published estimates of parent-reported ADHD diagnosis, by state and relevant demographics (2). The objective of this study was to estimate 2007 rates of medication treatment of ADHD, by state and sex-stratified age.

## Methods

The National Survey of Children’s Health (NSCH) is a national, random-digit–dialed landline telephone survey of approximately 30 minutes used to characterize the physical, behavioral, and emotional health and health experiences of American children. Conducted twice, the 2007–2008 NSCH had an overall response rate of 46.7%, which incorporates a cooperation rate of 66.0% (5). Nonresponse adjustments were made to the sampling weights to reduce nonresponse bias.

Parents and guardians reported whether they had ever been told by a doctor or health care professional that their child (aged 2-17 y) had attention-deficit disorder or ADHD. Respondents also reported whether the child currently had the disorder. If respondents reported a current diagnosis, they were asked whether the child was currently taking medication for ADHD. We used these data to estimate the percentage of children with current ADHD who were taking medication for ADHD and the rate of medicated ADHD among all US children, calculated by dividing the number of children who were currently taking medication for ADHD by the number of those who had valid responses (ie, those who responded either yes or no) to the current ADHD diagnosis question. We calculated national, state-based, and sex-by-age rates of these 2 indicators.

We restricted the data to 73,122 children aged 4 to 17 years for whom we had valid data on sex and ADHD diagnosis. We incorporated design elements (strata and nonresponse-adjusted weight variables) using SUDAAN version 10.0 (RTI International, Research Triangle Park, North Carolina) to account for the complex survey design and percentages of children taking medication for ADHD treatment by state and sex-stratified age. We compared the prevalence of ADHD medication by sex at each age by using χ^2^ tests and Wald *F* tests.

## Results

Of US children aged 4 to 17 years, 4.1 million (7.2%) had a current ADHD diagnosis in 2007, and approximately 2.7 million were taking ADHD medication. The percentage of children with current ADHD and taking ADHD medication was 4.8% (Table), which represents 66.3% of those with a current ADHD diagnosis.

**Table:** Parent-Reported Attention-Deficit/Hyperactivity Disorder (ADHD) (Current) and ADHD Medication Treatment Among Children Aged 4–17 Years, National Survey of Children’s Health, 2007–2008

State	US Census Region^a^	Children With a Current ADHD Diagnosis, % (95% CI)	Children Taking Medication for ADHD, % (95% CI)	Children With ADHD Taking Medication for ADHD, % (95% CI)
**United States**	National	7.2 (6.8–7.7)	4.8 (4.4–5.1)	66.3 (63.0–69.4)
Nevada	West	3.7 (2.3–5.7)	1.2 (0.7–2.1)	33.3 (18.0–53.1)
California	West	4.6 (3.0–7.1)	2.3 (1.3–4.0)	49.1 (28.2–70.3)
Alaska	West	6.0 (4.4–8.1)	2.4 (1.6–3.7)	40.7 (27.1–55.9)
Hawaii	West	4.5 (3.3–6.1)	2.5 (1.7–3.8)	56.6 (40.3–71.6)
New Mexico	West	4.3 (3.1–5.8)	2.9 (1.9–4.2)	67.2 (52.2–79.4)
Utah	West	4.8 (3.5–6.7)	3.1 (2.0–4.8)	65.2 (48.6–78.8)
Washington, DC	South	5.8 (4.4–7.5)	3.2 (2.2–4.8)	56.4 (43.0–68.9)
Texas	South	4.8 (3.2–7.0)	3.4 (2.2–5.4)	71.9 (50.8–86.4)
Illinois	Midwest	4.8 (3.6–6.3)	3.5 (2.5–4.9)	74.5 (61.3–84.3)
Arizona	West	5.9 (4.4–7.9)	3.5 (2.4–5.2)	60.3 (45.4–73.5)
Connecticut	Northeast	5.5 (4.3–7.0)	3.6 (2.6–4.9)	65.5 (52.9–76.2)
New Jersey	Northeast	6.9 (5.2–9.1)	3.6 (2.5–5.4)	52.9 (38.1–67.2)
Montana	West	6.3 (4.9–8.1)	3.7 (2.7–5.2)	59.5 (46.1–71.6)
Colorado	West	5.2 (3.7–7.2)	3.8 (2.5–5.6)	73.0 (56.3–85.0)
New York	Northeast	7.5 (5.6–10.0)	3.9 (2.6–5.8)	52.4 (37.3–67.1)
Washington	West	6.7 (4.9–9.2)	4.1 (2.8–6.0)	61.3 (44.4–75.9)
Vermont	Northeast	7.8 (6.1–10.1)	4.2 (3.0–5.8)	53.2 (39.9–66.1)
Idaho	West	6.6 (5.1–8.6)	4.2 (3.1–5.7)	63.1 (49.1–75.2)
Nebraska	Midwest	6.5 (4.7–8.8)	4.3 (2.9–6.3)	66.9 (49.5–80.6)
South Dakota	Midwest	6.6 (5.2–8.5)	4.5 (3.3–6.1)	67.5 (55.2–77.8)
Oregon	West	6.8 (5.1–9.0)	4.5 (3.1–6.6)	66.4 (52.6–77.9)
Wyoming	West	6.7 (5.1–8.7)	4.5 (3.3–6.3)	67.9 (54.2–79.1)
Minnesota	Midwest	6.5 (5.0–8.4)	4.6 (3.4–6.2)	71.1 (57.0–82.1)
Maine	Northeast	7.3 (5.8–9.2)	4.6 (3.4–6.2)	63.9 (51.9–74.3)
Virginia	South	8.2 (6.6–10.2)	4.8 (3.6–6.4)	58.6 (47.0–69.3)
Michigan	Midwest	8.2 (6.4–10.4)	5.1 (3.8–6.7)	62.5 (49.2–74.1)
North Dakota	Midwest	7.5 (6.0–9.3)	5.1 (3.9–6.6)	68.3 (56.9–77.9)
Georgia	South	6.9 (5.3–8.9)	5.2 (3.9–7.0)	76.1 (63.3–85.5)
New Hampshire	Northeast	7.2 (5.7–9.0)	5.3 (4.1–6.9)	74.3 (63.0–83.1)
Kansas	Midwest	7.6 (6.0–9.5)	5.5 (4.2–7.1)	73.0 (60.7–82.6)
Pennsylvania	Northeast	7.9 (5.7–10.8)	5.6 (3.7–8.4)	70.3 (55.4–81.9)
Ohio	Midwest	9.4 (7.2–12.2)	5.7 (4.1–7.9)	61.1 (46.4–74.1)
Massachusetts	Northeast	8.0 (6.1–10.3)	5.7 (4.2–7.8)	71.8 (57.7–82.6)
Mississippi	South	7.3 (5.8–9.1)	5.8 (4.4–7.5)	79.0 (69.1–86.4)
Oklahoma	South	8.4 (6.5–10.7)	5.8 (4.3–7.8)	69.2 (56.1–79.8)
Wisconsin	Midwest	7.6 (6.0–9.7)	5.8 (4.4–7.7)	76.4 (65.1–85.0)
Iowa	Midwest	7.6 (5.7–10.7)	5.9 (4.3–8.1)	78.2 (61.7–88.9)
Maryland	South	9.1 (7.2–11.5)	6.1 (4.7–7.8)	66.5 (53.0–77.8)
Florida	South	8.9 (6.4–12.4)	6.2 (4.0–9.3)	69.1 (51.7–82.4)
Tennessee	South	8.7 (6.9–10.9)	6.2 (4.7–8.2)	71.8 (60.3–80.9)
Missouri	Midwest	8.6 (7.0–10.5)	6.7 (5.3–8.4)	78.3 (67.6–86.2)
Indiana	Midwest	9.3 (7.4–11.8)	6.8 (5.2–8.9)	73.2 (60.1–83.2)
South Carolina	South	9.5 (7.5–11.8)	6.9 (5.3–8.9)	73.0 (60.6–82.6)
Rhode Island	Northeast	9.4 (7.4–11.7)	7.1 (5.4–9.3)	75.8 (64.8–84.1)
Kentucky	South	10.2 (8.4–12.2)	7.1 (5.7–8.9)	70.4 (60.8–78.5)
Alabama	South	9.7 (7.7–12.1)	7.2 (5.5–9.3)	74.8 (63.3–83.6)
Delaware	South	10.9 (8.9–13.4)	7.3 (5.6–9.4)	66.8 (55.7–76.4)
Arkansas	South	10.9 (8.9–13.2)	7.5 (5.9–9.5)	69.2 (58.2–78.3)
West Virginia	South	11.1 (9.1–13.6)	7.8 (6.1–9.8)	70.0 (58.6–79.4)
Louisiana	South	11.7 (9.3–14.6)	8.3 (6.4–10.6)	70.7 (57.8–81.0)
North Carolina	South	12.8 (10.5–15.7)	9.4 (7.3–12.0)	74.4 (64.6–82.2)

Abbreviation: CI, confidence interval.

a 2007 economic census regions (6) were used to group states into geographic regions.

Among children aged 4 to 17 years, current ADHD was associated with increasing age and male sex (Figure); rates were significantly higher among boys aged 5 to 17 years (χ^2^ test, *P* < .05). Medication treatment rates by age were significantly different as a function of sex (age-by-sex interaction: *F*_13,73,121_ = 2.08; *P* = .01); rates of medication treatment increased during early childhood for both sexes, leveled off among older girls, and decreased among teenaged boys. Medication rates were higher among boys than girls at every age.

**Figure:**
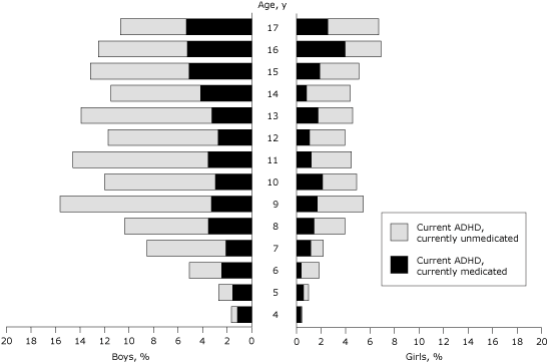
Parent-reported current attention-deficit/hyperactivity disorder (ADHD) and ADHD medication treatment among US children, by age and sex, National Survey of Children’s Health, 2007–2008. **Table:** 

Age, y	Boys		Girls	
	Current ADHD, Unmedicated, %	Current ADHD, Medicated, %	Current ADHD, Unmedicated, %	Current ADHD, Medicated, %
4	1.20	0.48	0.36	0.06
5	1.59	1.13	0.56	0.41
6	2.49	2.63	0.38	1.45
7	2.14	6.44	1.16	1.01
8	3.57	6.83	1.42	2.52
9	3.33	12.34	1.69	3.77
10	2.99	9.04	2.12	2.78
11	3.58	11.05	1.20	3.28
12	2.78	8.98	1.06	2.88
13	3.28	10.68	1.75	2.83
14	4.21	7.31	0.80	3.57
15	5.15	8.06	1.90	3.19
16	5.28	7.26	3.96	2.92
17	5.37	5.38	2.55	4.15

The lowest state-based rates of medicated ADHD were documented in 5 Western states (Nevada, California, Alaska, Hawaii, and New Mexico); the highest rates were documented in 5 Southern states (North Carolina, Louisiana, West Virginia, Arkansas, and Delaware). The percentage of children with current ADHD taking ADHD medication was lowest in Nevada, Alaska, and California and highest in Iowa, Missouri, and Mississippi. The national rate of medication treatment among children with current ADHD was 66.3% (95% confidence interval, 63.0-69.4); state-based rates ranged from 33% in Nevada to 79% in Mississippi.

## Discussion

Nearly 3 million school-aged children were taking medication for ADHD in 2007; this finding is similar to that of a recent analysis of claims data, indicating that 2.8 million (3.5%) US children aged 18 years or younger were taking a stimulant medication in 2008 (7). Because of changes in who was asked about current medication treatment of ADHD across the 2 surveys (parents of those ever diagnosed in 2003 vs parents of those with a current diagnosis in 2007), making direct comparisons of the 2 percentages (4.8% in 2007 and 4.3% in 2003) is not possible (8). However, the 2007 survey design change more likely underestimates, rather than overestimates, the total number of children taking ADHD medications. Because the 2007 medication treatment question was only asked about children reported to have a current ADHD diagnosis, there may have been children taking medication for ADHD who did not meet criteria for ADHD at the time of the survey, resulting from either successful or inappropriate treatment. A limitation of this study is that the NSCH collects parent-reported data; despite the estimates in this report being consistent with those generated from claims data sources, the ADHD survey questions have not been formally validated.

The differences in the sex-stratified and state-based rates of medicated ADHD may serve to inform state-based policy and programmatic efforts. Specifically, girls and boys with ADHD may have different medication needs over time; future studies should investigate the potential association between sex differences in medication use and ADHD subtypes. State-based variation in ADHD medication treatment supports reports by the Food and Drug Administration of uneven product distribution of common ADHD medications, contributing to ADHD medication shortages in certain states (9). Variability in access to preventive and mental health services as well as provider characteristics could account for some of the geographic variation noted in this study. Future state-based investigations will be helpful in identifying the factors contributing to the patterns of ADHD prevalence and medication treatment by state and region.
