# Erratum, Vol. 10, January 24 Release

**DOI:** 10.5888/pcd10.120073e

**Published:** 2013-02-14

In “State-Based and Demographic Variation in Parent-Reported Medication Rates for Attention-Deficit/Hyperactivity Disorder, 2007–2008,” the stacked bars in the Figure were incorrectly shaded and did not correspond with the data provided by the authors. The corrections were made to our website on February 5, 2013, and appear online at www.cdc.gov/pcd/issues/2013/12_0073.htm. We regret any confusion or inconvenience this error may have caused.

